# An evaluation of gastric adenocarcinoma-associated CircRNAs based on microarray meta-analysis and ceRNA networks

**DOI:** 10.1016/j.tranon.2022.101611

**Published:** 2022-12-29

**Authors:** Liora Yesharim, Saeed Talebi, Marzieh Mojbafan, Mahdi Alemrajabi, Shahram Teimourian

**Affiliations:** a Department of Medical Genetics, School of Medicine, Iran University of Medical Sciences, Tehran, Iran; b Department of General Surgery, School of Medicine, Firoozgar General Hospital, Iran University of Medical Sciences, Tehran, Iran

**Keywords:** Circular RNA, Gastric adenocarcinoma, qRT-PCR, Microarray, ceRNA

## Abstract

Gastric cancer is the fourth leading cause of cancer-related mortality and one of the most commonly diagnosed malignancies worldwide. Gastric adenocarcinoma (GAC) accounts for the majority of gastric cancer cases. Circular RNAs (circRNAs) have been shown to be associated with carcinogenesis and cancer progression. This research aims to investigate GAC-associated circRNAs and the underlying mechanisms of circRNA-miRNA-mRNA networks in the development and progression of GAC. Differentially expressed miRNAs and mRNAs (DEMs and DEGs) were identified in Gene Expression Omnibus (GEO) microarray datasets using the R package Limma. A microarray meta-analysis was performed to identify potential GAC-associated circRNAs with high statistical power, resulting in 13 up-regulated and 19 down-regulated circRNAs. CircRNA-miRNA-mRNA networks were constructed by combining predicted and experimentally validated databases and considering differentially expressed miRNAs and mRNAs. The constructed ceRNA networks revealed the potential regulatory effect of *hsa_circ_0002019* and *hsa_circ_0074736* on key survival-related genes. The expression levels of these two circRNAs were measured in plasma samples from GAC patients and healthy controls using SYBR Green-based real-time PCR. Axon guidance, cellular senescence, AGE-RAGE signaling pathway in diabetic complications, and AMPK signaling pathway were among the major significant (*P*-value <0.05) enriched pathways of "main mRNAs" in the constructed ceRNA networks. In conclusion, we identified strongly correlated circRNAs and their likely mechanisms of action in GAC, which may improve the knowledge of regulatory networks underlying GAC formation and contribute to developing better strategies for early diagnosis, prognosis, and treatment.

## Highlights

• A microarray meta-analysis revealed 32 dysregulated circRNAs in gastric adenocarcinoma.
• *hsa_circ_0002019* & *hsa_circ_0074736* showed no significant fold change in plasma.
• *hsa_circ_0002019* & *hsa_circ_0074736* regulate 4 mRNAs affecting gastric cancer survival.

## Graphical abstract

**Figure:**
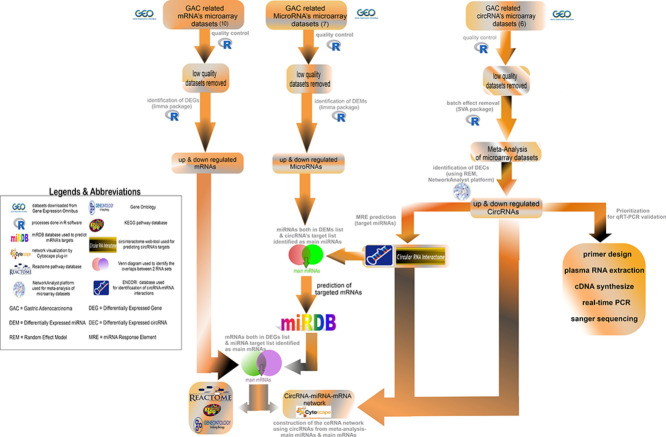

## Introduction

In 2020, there were an estimated 19.3 million and 9.9 million new cancer cases and cancer-related deaths, respectively. In particular, gastric cancer was responsible for over one million new cases in 2020 and is the fourth leading cause of cancer-related mortality [1]. Gastric adenocarcinoma (GAC) accounts for more than 90% of all gastric cancer cases. Despite all the advances, the overall survival rate of patients with GAC is low. This is mainly because most cases are diagnosed at advanced stages, and current treatment strategies are no longer effective at advanced stages [2,3]. Thus, it is critical to implement novel diagnostic and prognostic biomarkers and effective therapeutic targets and techniques to enhance patient survival and quality of life.

Endoscopy with biopsy is currently the gold standard for diagnosing gastric cancer [4]. However, there are certain limitations to this strategy. Biopsies may not give efficient information on the tumor profile due to the heterogeneous nature of tumors. Furthermore, these procedures are invasive and do not identify cancerous masses in their early stages. Meanwhile, liquid biopsies like plasma can be obtained in a minimal-invasive manner and clearly identify the full characteristics of the tumor even in its early stages and provide the possibility of multiple sampling to monitor the disease's condition [5].

Circular RNAs (CircRNAs) are covalently closed single-stranded RNA molecules, mainly formed by the process of "backsplicing" from precursor mRNAs. They have no free ends, and as a result, they are highly stable due to their resistance to exonucleases [6]. CircRNAs are conserved, have tissue-specific expression, and play various roles in the cell [7]. Some circRNAs can regulate RNA transcription, can function as miRNA sponges, protein scaffolds, and protein transporters, and some can be translated into proteins [8].

CircRNAs have attracted a great deal of attention as cancer biomarkers in recent years. The high stability of circRNAs, especially in body fluids such as plasma, and their potential ability to affect cancer-related pathways have made them suitable biomarkers for managing cancer in various ways, including diagnosis, prognosis, or response to treatment [9]. Recent research showed that up-regulation of *hsa_circ_0005092* and *hsa_circ_0002647* positively correlated with recurrence-free survival and overall survival in GAC patients [10]. Another study found significant downregulation of *hsa_circ_0021087* and *hsa_circ_0005051* in GC tissues, cells, and plasma, suggesting that the dual-circular RNA signature might be used as a non-invasive diagnostic biomarker for GC [11]. Additionally, CircRNAs can potentially be used to treat various diseases, including cancer [12]. Knocking down oncogenic circRNAs would be beneficial to suppress cell proliferation, metastasis, and other cancer hallmarks [13,14]. Furthermore, it is also feasible to take advantage of circRNA sponge capabilities to efficiently sponge critical miRNAs involved in pathogenesis by overexpressing specific circRNAs [2,8].

This study was carried out due to the utility and necessity of finding novel biomarkers that can be obtained in plasma samples and the need for new therapeutic targets. CircRNAs were chosen for this purpose because of their unique features and regulatory functions in the cell. A meta-analysis of circRNA's microarray datasets was performed. In addition, a total of 200 miRNA microarray samples and 310 mRNA microarray samples were also analyzed to identify differentially expressed miRNAs and mRNAs. CircRNA-miRNA-mRNA networks were constructed by combining predicted and experimentally validated databases and considering differentially expressed miRNAs and mRNAs. The result of our study highlights GAC's strongly associated differentially expressed circRNAs which may serve as effective biomarkers and drug targets for GAC and highlights possible underlying mechanisms of ceRNA networks in the development and progression of GAC.

## Materials & methods

### Microarray data selection and identifying differentially expressed RNAs

GEO (https://www.ncbi.nlm.nih.gov/geo/) database was searched for mRNA, miRNA, and circRNA microarray expression profiles of samples from gastric adenocarcinoma. A total of 23 datasets were downloaded and imported into R software (version 3.6.1) for further analysis, comprising ten mRNA, seven miRNA, and six circRNA microarray expression profiles. The quality of the 23 datasets was initially assessed using boxplots, PCA (Principal Component Analysis), and heat maps. Datasets with acceptable quality were chosen for further analysis.

The R package "limma" [15] was used for quantile normalization and differential expression analysis on datasets of acceptable quality. The differentially expressed miRNAs (DEMs) and differentially expressed mRNAs (DEGs) were screened with the cut-off of 0.05 Benjamini-Hochberg's adjusted *P*-value and |log2FC|>1. The miRBaseConverter Shiny app was used to convert all DEMs to the latest version of miRNA names [16].

The NetworkAnalyst web interface was used for the meta-analysis of circRNA microarray datasets [17,18]. The ComBat procedure was used to adjust the batch effect of pre-normalized and log-transformed datasets. The combined effect size technique of meta-analysis (which integrates two fundamental methodologies, the random effect model (REM) and the fixed effect model (FEM)), was used to identify differentially expressed circRNAs (DECs) between gastric cancer and non-cancer samples. Since Cochrane's Q test revealed a considerable deviation from a chi-squared distribution, the random effect model (REM) was chosen over the fixed effect model (FEM). DECs have been selected with the same cut-off threshold as DEMs and DEGs.

### Construction of the CircRNA-miRNA-mRNA regulatory network

**Fig. 1:**
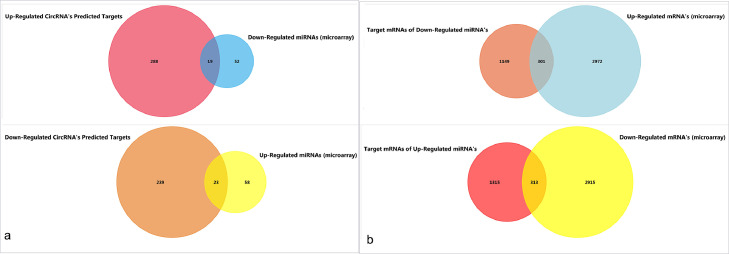
Venn diagrams showing (a) the overlap between predicted targets of DECs and dysregulated miRNAs identified by microarray analysis and (b) the overlap between predicted targets of main miRNAs and DEGs.

Certain circRNAs can operate as competitive endogenous RNA (ceRNA) of microRNAs. CircRNAs competitively bind to miRNA response elements (MREs) (a phenomenon known as sponging) and thus reduce the chance of mRNAs interacting with miRNAs. To find possible regulatory networks, we first select the miRNA targets of the circRNAs identified in the meta-analysis by CircInteractome [19] and ENCORI [20]. CircInteractome uses the TargetScan algorithm to predict target miRNAs, and ENCORI employs Ago-CLIP-Seq supported data for circRNA-miRNA interactions. Only miRNAs that were replicated in at least three CLIP-Seq experiments in ENCORI were taken into account. One of the essential aspects of assuming the circRNA's sponging role is the dysregulation of target miRNAs in the disease condition. As a result, the proportion of predicted target miRNAs that showed overlap (Fig. 1a) with DEMs are regarded as “main miRNAs”.

The miRDB online database [21] -which uses a machine learning framework for genome-wide miRNA target prediction- was used to find genes targeted by key miRNAs. Predicted mRNAs with a score greater than 90 were downloaded. The mRNAs included in both the list of up/down-regulated DEGs and the miRNA's target mRNAs were also identified using Venn diagrams (Fig. 1b). The Circ-miRNA-mRNA networks were constructed using Cytoscape (version 3.8.9).

### Gene ontology and pathway enrichment analysis

**Fig. 2:**
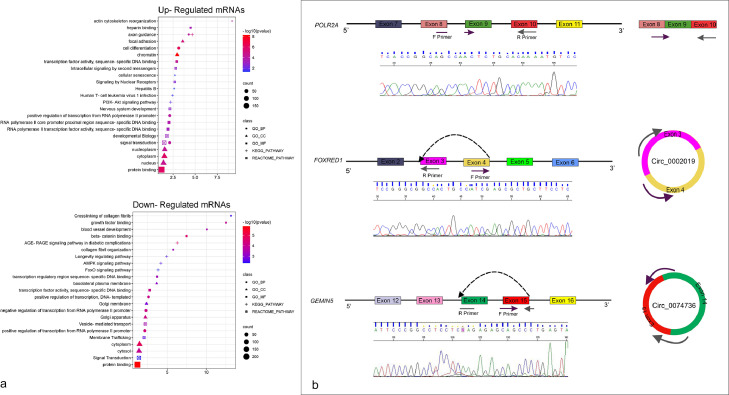
(a) The most enriched GO and pathways (KEGG & REACTOME) terms for the up-regulated and down-regulated mRNAs involved in ceRNA networks. (b) A schematic showing the position of the designed primers of the reference gene and circRNAs examined in this study by real-time PCR and Sanger sequencing.

Enrichment studies of Gene Ontology (GO), Kyoto Encyclopedia of Genes and Genomes (KEGG), and REACTOME pathway were done for the overlap mRNAs using the online tool Database for Annotation, Visualization, and Integrated Discovery (DAVID) [22]. The most enriched gene ontology terms are shown in (Fig. 2a). Statistical significance was defined as a P-value of 0.05.

### CircRNAs prioritization and primer design

CircRNAs obtained from Meta-analysis of microarray datasets were prioritized according to the following factors: higher LogFC, suitable size, and sequence for primer design. Additionally, circRNAs that have not yet been investigated were prioritized according to literature reviews. CircInteractome [19] and primer3 [23] web tools were used to design divergent primers for circRNAs. NCBI primer blast and circPrimer [24] were utilized to evaluate the specificity of primers. Primer sequences are shown in Table 1.

**Table 1:** Sequence of primers used in this study.

CircRNA/Gene	Primer Sequence (5’-3’)	Primer Length	GC%	TM	Product Length
*(POLR2A)RPII*	—	—	—	—	170
	GACCGGCTATAAGGTGGAACG	21	57	60.5	
	CGTCAAAGTCTGCATTGTACGG	22	50	60.1	
*hsa_circ_0074736*					161
	CCTGTGCAATTTCCGAGGAC	20	55	59.2	
	ACTCAGGGCTGCTCTCTTGA	20	55	60.5	
*hsa_circ_0002019*					210
	GTACCTGGCCGTAGTCGATG	20	60	59.9	
—	GCTGGCTGAAAAGAGGGAGA	20	55	59.6	—

### Plasma samples and RNA extraction

Blood samples were obtained from 18 patients diagnosed with gastric adenocarcinoma and 15 age and sex-match healthy individuals in EDTA tubes. All participants signed the informed consent form approved by the Research Ethics Committee of the Iran University of Medical Sciences (IR.IUMS.FMD.REC.1399.561). Blood samples were centrifuged at 150 g (∼900 RPM) for 20 min and two-thirds of the upper plasma phase was transferred to cryovials and frozen in liquid nitrogen and stored at -80 °C until RNA extraction. Total RNA was extracted from 250 µl plasma using Ribo Ex LS (Geneall-South Korea) reagent. The study excluded samples from people with metastasis to other tissues, turbid plasma, or poor RNA/cDNA quality (double-checked).

### Reverse transcription, real-time PCR, and statistical analysis

The cDNAs were obtained by reverse transcription from 50 ng of total RNA with AddScript cDNA synthesis kit (AddBio, Korea) using random hexamers. Real-time PCR was performed with the Step-One Plus real-time PCR System (Applied Biosystems) using the SYBR Green qPCR master mix (Yekta Tajhiz Azma, IRAN). The PCR assay was conducted at an initial denaturation step at 95 °C for 3 min, followed by 50 cycles at 95 °C for 5 s and 60 °C for 10 s, and 72 °C for 10 s. The melting curve analysis and sanger sequencing were done to confirm amplification's specificity. All Real-time PCR experiments were performed in duplicates. Fold differences between cancer samples versus normal samples were calculated using the E^−ΔΔCt^ method with *POLR2A (RPIIA)* mRNA as an internal control. As circRNAs are relatively low-expressing RNAs, the best approach is to choose the reference gene with the same expression level. *POLR2A* is considered a low-expressing reference gene (median CT >30). Additionally, POLR2A was shown to be the best reference gene for RNA transcription analysis using qRT PCR [25]. LinregPCR software (v 2012.1) was used to calculate the mean efficiency of each target. The relative expression software tool (REST 2009 v2.0.13) [26] was used to determine the statistical significance of expression ratios using a pairwise fixed reallocation randomization test (2000 randomizations).

### Possible prognostic value of selected CircRNAs

The following steps were adopted to evaluate whether the selected cirRNAs have a prognostic value in patients with gastric adenocarcinoma.

GEPIA was used to download 100 mRNAs that showed the most significant effect on the mean overall survival of patients with GAC. Data from GTEx and TCGA projects are collected and processed in this web server, and information such as differential expression and patient survival analysis are available [27].

ENCORI (no cut-off admitted) and CircInteractome were used to identify the target miRNAs of the two selected circRNAs *(hsa-circ_0002019* and *hsa_circ_0074736*). Target mRNAs of the resulting miRNAs (with target scores greater than 90) were chosen from miRDB. Following that, the Cytoscape-generated network was used to assess the overlap between the target mRNAs from miRDB and the most differential survival genes from GEPIA.

## Results

### Differentially expressed RNAs identification

Boxplots, PCA plots, and heat maps were used to assess the quality of 23 chosen mRNA, miRNA, and circRNA microarray databases. Five mRNA, five miRNA, and four circRNA microarray datasets were chosen for further investigation (a list of the selected datasets is shown in Table 2).

**Table 2:** Microarray datasets with acceptable quality which used in this study.

GEO Accession Number	Platform	RNA Type	RNA Type	Sample Size (Cancer/Normal)
GSE78092	Arraystar GPL21485	circRNA	circRNA	3/3
GSE83521	Arraystar GPL19978	circRNA	circRNA	6/6
GSE89143	Arraystar GPL19978	circRNA	circRNA	3/3
GSE93541	Arraystar GPL19978	circRNA	circRNA	3/3
GSE26595	Illumina GPL8179	microRNA	microRNA	52/8
GSE23739	Agilent GPL7731	microRNA	microRNA	40/40
GSE28700	Agilent GPL9081	microRNA	microRNA	22/22
GSE67354	GenoExplorer GPL19952	microRNA	microRNA	5/5
GSE78091	miRCURY LNA GPL21439	microRNA	microRNA	3/3
GSE33335	Affymetrix GPL5175	mRNA	mRNA	25/25
GSE54129	Affymetrix GPL570	mRNA	mRNA	111/21
GSE118916	Affymetrix GPL15207	mRNA	mRNA	15/15
GSE19826*	Affymertix GPL570	mRNA	mRNA	12/15
GSE13911*	Affymertix GPL570	mRNA	mRNA	32/39

*The following samples were identified as outliers by heat maps and PCA plots and removed from GSE19826 (GSM495051, GSM495063, GSM495071, GSM495072) and GSE13911 (GSM350427, GSM350469, GSM450475, GSM450477, GSM450479).

The DEGs and DEMs were identified in each dataset after the raw data of mRNA, and miRNAs were log2 transformed, quantile normalized, and outliers were removed if necessary. In the five miRNA and five mRNA microarray datasets, 81 up-regulated and 71 down-regulated miRNAs and 3273 up-regulated, and 3228 down-regulated mRNAs (duplicate removed) were filtered out based on Benjamini-Hochberg's adj.*P-value* 0.05 and |LogFC| > 1.

The four circRNA datasets were utilized in NetworkAnalyst for REM-based meta-analysis. CircRNA's datasets were also log2 transformed and quantile normalized, and the batch effect between the four datasets was removed using ComBat beforehand. A total of 32 circRNA were found to be differently expressed between cancer and normal samples, including 13 up-regulated and 19 down-regulated circRNA (Table 3).

**Table 3:** A list of the differentially expressed circRNAs identified by microarray mete-analysis.

*Circbase ID*	*Alias*	*CombinedES*	*P value*	*Host Gene name*	*Splice Length*	
*Down-Regulated circRNAs*						
*hsa_circ_0007991*	*hsa_circRNA_100084*	-2.65	0.00071	*EIF4G3*	301	
*hsa_circ_0067934*	*hsa_circRNA_103510*	-2.38	0.00132	*PRKC1*	170	
*hsa_circ_0048607*	*hsa_circRNA_102417*	-2.30	0.00152	*CHAF1A*	857	
*hsa_circ_0013048*	*hsa_circRNA_100269*	-2.08	0.00252	*LPHN2*	387	
*hsa_circ_0003763*	*hsa_circRNA_103442*	-2.06	0.00299	*GSK3B*	286	
*hsa_circ_0021087*	*hsa_circRNA_100754*	-1.99	0.00303	*LMO1*	340	
*hsa_circ_0074736*	*hsa_circRNA_103994*	-1.93	0.00368	*GEMIN5*	312	
*hsa_circ_0065214*	*hsa_circRNA_103348*	-1.90	0.00414	*SCAP*	915	
*hsa_circ_0000940*	*hsa_circRNA_102567*	-1.82	0.04641	*MARK4*	103	
*hsa_circ_0004599*	*hsa_circRNA_103808*	-1.78	0.00835	*DROSHA*	308	
*hsa_circ_0010501*	*hsa_circRNA_100089*	-1.74	0.01054	*ECE1*	179	
*hsa_circ_0030793*	*hsa_circRNA_101296*	-1.73	0.00963	*FGF14*	215	
*hsa_circ_0008274*	*hsa_circRNA_101287*	-1.63	0.01766	*UGGT2*	244	
*hsa_circ_0006088*	*hsa_circRNA_104931*	-1.57	0.02433	*SPTAN1*	503	
*hsa_circ_0038649*	*hsa_circRNA_101768*	-1.52	0.02222	*PRKCB*	777	
*hsa_circ_0000434*	*hsa_circRNA_000552*	-1.52	0.02569	*KIA1033*	110	
*hsa_circ_0088045*	*hsa_circRNA_104872*	-1.49	0.02569	*SUSD1*	303	
*hsa_circ_0070382*	*hsa_circRNA_103691*	-1.48	0.02569	*AFF1*	169	
*hsa_circ_0034510*	*hsa_circRNA_101474*	-1.38	0.04641	*THBS1*	219	
*Up-Regulated circRNAs*						
*hsa_circ_0001443*	*hsa_circRNA_000711*	1.40	0.04554	*MAML3*	131	
*hsa_circ_0008856*	*hsa_circRNA_100635*	1.40	0.04041	*TSPAN14*	318	
*hsa_circ_0006827*	*hsa_circRNA_100438*	1.44	0.03345	*chr1: 207989273-207990759 strand: -*	1486	
*hsa_circ_0037969*	*hsa_circRNA_101711*	1.48	0.03345	*PARN*	785	
*hsa_circ_0031250*	*hsa_circRNA_101320*	1.49	0.02569	*PRMT5*	327	
*hsa_circ_0079492*	*hsa_circRNA_104319*	1.60	0.01597	*BZW2*	416	
*hsa_circ_0080000*	*hsa_circRNA_104357*	1.71	0.01155	*DBNL*	244	
*hsa_circ_0007763*	*hsa_circRNA_101689*	1.82	0.00835	*NAA60*	246	
*hsa_circ_0008253*	*hsa_circRNA_103089*	1.85	0.01763	*ATP9A*	259	
*hsa_circ_0007767*	*hsa_circRNA_100904*	1.87	0.04584	*ALG8*	409	
*hsa_circ_0050102*	*hsa_circRNA_102485*	1.97	0.00963	*PGPEP1*	7064	
*hsa_circ_0019054*	*hsa_circRNA_100641*	2.09	0.00252	*ATAD1*	308	
*hsa_circ_0002019*	*hsa_circRNA_100984*	2.13	0.00252	*FOXRED1*	230	

### CircRNA-miRNA-mRNA regulatory networks and functional enriched terms

The 32 DECs identified by microarray meta-analysis were used for miRNA target prediction by CircInteractome and ENCORI. 307 and 262 non-redundant miRNAs were identified as targets for up and down-regulated circRNAs, respectively. Among these targets, 23 overlap with up-regulated miRNAs, and 19 overlap with down-regulated miRNAs.

**Fig. 3:**
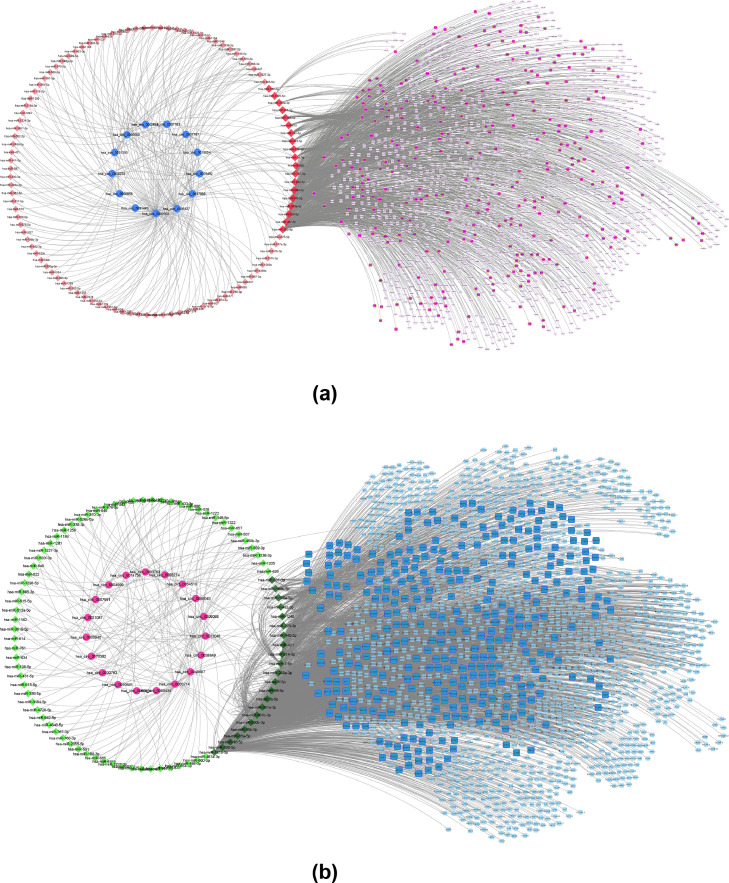
Up-regulated (a) and (b) down-regulated circRNAs mediated ceRNA networks. DECs are shown with circles. The target miRNAs are shown with diamond shapes and the target mRNAs are shown with squares. Main miRNAs and mRNAs (miRNAs and mRNAs that appear in both the list of predicted targets and the list of differentially expressed miRNAs and mRNAs) are shown larger and bolder.

The miRDB dataset was used to determine the target mRNAs for each overlapping miRNAs (main miRNAs). A total of 643 overlaps with microarray DEGs were found, including 301 overlaps with up-regulated mRNAs and 342 overlaps with down-regulated mRNAs (main mRNAs). The Cytoscape generated CircRNA-miRNA-mRNA networks are visualized in Fig. 3.

The 301 overlappings up-regulated and 342 overlapping down-regulated mRNAs (main mRNAs) were used to perform GO, KEGG, and REACTOME pathway functional enrichment analyzes. Fig. 2a shows the top five highly enriched terms in each category. Up-regulated genes were mostly involved in axon guidance, cellular senescence, and PI3K-Akt signaling pathway. The down-regulated genes were mainly associated with the AGE-RAGE signaling pathway in diabetic complications, the AMPK signaling pathway, and FoxO signaling pathway.

### *hsa_circ_0002019* and *hsa_circ_0074736* in plasma

**Fig. 4:**
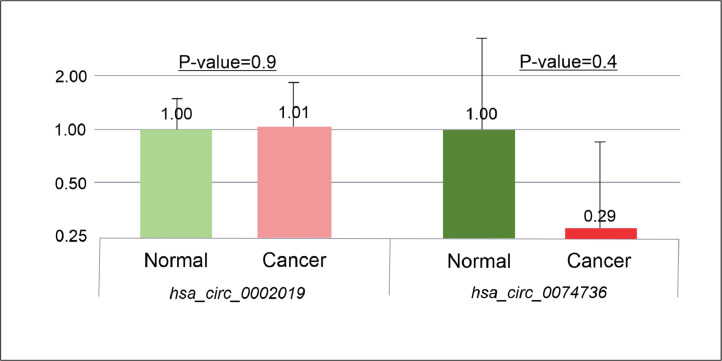
The expression levels of hsa_circ_0002019 and hsa_circ_0074739 in plasma samples of GAC patients and healthy controls.

A microarray meta-analysis of circRNAs revealed 32 dysregulated circRNAs in GAC. Using real-time PCR, the expression levels of one up-regulated circRNA *(hsa_circ_0002019)* and one down-regulated circRNA (*hsa_circ_0074736)* were evaluated in plasma samples. Although the other 6 circRNAs (*hsa_circ_0007991* [28], *hsa_circ_0067934* [28], *hsa_circ_0048607* [29]*, hsa_circ_0013048* [30]*, hsa_circ_0003763*(30)*, hsa_circ_0021087* [11] had higher log2FC in the down-regulated group, *hsa_circ_0074736* was chosen because this circRNA has not been investigated before. The results showed no significant (*P*-value < 0.05) changes in the expression levels of these two circRNAs (Fig. 4).

### *hsa_circ_0002019* and *hsa_circ_0074736* as potential prognostic biomarkers

**Fig. 5:**
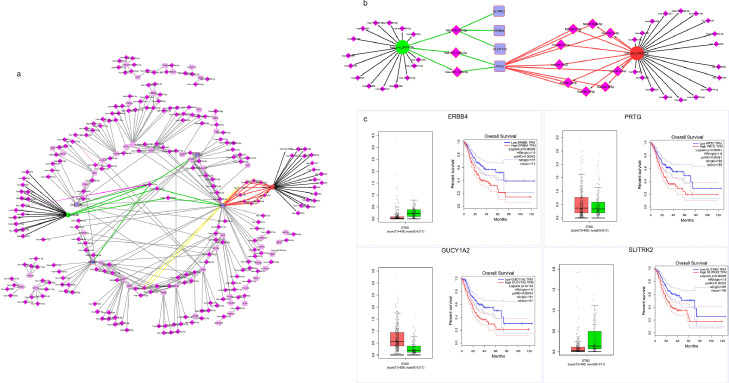
(a) Predicted ceRNA network regulated by hsa-circ_0002019 and hsa_0074736; showing their interaction with *ERRB4, PRTG, GUCY1A2* and *SLITRK2* mRNAs, which have the greatest impact on mean overall survival of GAC patients. (b) Subnetwork from part (a) showing the major interacting part more clearly. (c) Kapan-Meier survival plots and differential expression analysis of the four genes. The four genes showed to have the greatest impact on the mean overall survival of GAC patients despite not being differentially expressed considering |logFC| > 1 and *P*-value < 0.05 as cutoff values. (STAD= Stomach (gastric) Adenocarcinoma).

Using Cytoscape, a network was constructed between *hsa_circ_0002019* and *hsa_circ_0074736* and their target mRNAs mediated by predicted miRNAs (Fig. 5a). Interestingly, the findings revealed that *hsa_circ_0002019* and *hsa_circ_0074736* can influence four mRNAs with the most significant impact on mean overall survival: *SITRK2, ERBB4, GUCY1A2,* and *PRTG*. Fig. 5c shows the Kaplan-Meier plot of overall survival for these four genes in GAC patients with a log-rank *P*-value of 0.05 and a hazard ratio (HR) of 95%. The GEPIA Webtool was also used to evaluate the changes in expression levels of these four genes in 408 tumor tissues and 211 normal tissues. The results showed no significant difference in the expression of these genes between cancer and normal states. However, their overexpression in GAC patients is associated with lower overall survival.

## Discussion

Gastric cancer is the fourth leading cause of cancer deaths, accounting for about one million new cases in 2020. It is the leading cause of cancer death in men in some Asian countries, including Iran [1]. Adenocarcinomas account for the majority of gastric cancers. Despite advances in diagnosis and treatment, the 5-year survival rate for stomach cancer remains poor (less than 30 %) [3].

In the current clinical landscape, biopsy remains the gold standard as the diagnostic method for GAC [4]. However, the information provided by a biopsy is limited to a specific time and location and lacks the specificity needed to evaluate changes resulting from disease progression or response to treatment. As an alternative, liquid biopsies can be collected non-invasively or semi-invasively and detect all aspects of the tumor, even in its early stages [5].

CircRNAs were found suitable targets for detection in liquid biopsies such as plasma [31,32]. CircRNAs express differently in different tissues, are highly resistant to exonucleases because of the lack of a free end, and serve various roles in the cell. Some circRNAs can regulate RNA transcription [33] to operate as miRNA sponges, protein scaffolds, and protein transporters, and some can even be translated into proteins [8]. CircRNAs appear to be ideal biomarkers for cancer management and monitoring due to their high stability, particularly in body fluids such as plasma, and their potential ability to influence cancer-related pathways.

To identify GAC-related circRNAs, a meta-analysis of microarray datasets was carried out, yielding 32 DECs. DECs, DEMs, and DEGs were used to construct potential ceRNA networks involved in GAC. The mRNAs that were both differentially expressed and potential targets of key miRNAs (main mRNAs) were used for enrichment analysis to identify relevant enriched ontologies and pathways involved in GAC development.

The ceRNA network constructed considering the mRNAs with the greatest impact on mean overall survival of patients with GAC showed that *hsa_circ_0002019* and *hsa_circ_0074736* compete with four of the most important genes (*SLITRK2, ERBB4, GUCY1A2, PRTG*) associated with gastric cancer survival for 12 miRNA and thus can potentially regulate the expression of these four genes at the post-transcriptional level.

Although real-time PCR analysis of the expression of *hsa_circ_0002019* and *hsa_circ_0074736* in plasma from GAC patients and healthy controls did not reveal significant differences, this does not mean that these two circRNAs should no longer be considered in biomarker research. Contrary to popular belief, the difference in expression between normal and cancerous conditions does not help determine prognosis-related genes [34]. Instead, we suggest that a within-group comparison of gene expression in cancer patients might be a better approach.

To the best of our knowledge, this is the first time that the expression of *hsa_circ_0002019* and *hsa_circ_0074736* has been studied using real-time PCR in plasma. Based on the results obtained in this study, it seems that *hsa_circ_0002019* and *hsa_circ_0074736* might not be helpful as diagnostic biomarkers for GAC. However, bioinformatics research implies that they may be valuable as prognostic biomarkers upon further investigation.

This study has several limitations, which we would like to highlight and make recommendations for future research.

First of all, the results obtained in this study may be due to limits of detection of the techniques and materials used. Plasma samples yield a small amount of RNA, and detection of circRNAs expressed at relatively low levels—increases the difficulty and carries the risk of erroneous results. We believe that utilizing locked nucleic acid (LNA) primers instead of conventional primers can improve template binding strength and specificity, thereby increasing amplification success and sensitivity [35]. Examining the expression of circRNAs in the tissue samples and then in the plasma is one of the ways to ensure the proper functioning of materials (including primers) and to get a better insight into the limit of detection of the methods used. In addition, considering that digital droplet PCR (ddPCR) provides more accurate, reproducible, and statistically meaningful results when only a small amount of template is present [36], we suggest taking advantage of ddPCR rather than qPCR when doing expression analysis of circRNAs in liquid biopsies. We also believe enrichment of exosomes before RNA extraction would be beneficial. Another essential issue is that the limited sample size may have been the reason for not rejecting the null hypothesis (getting an insignificant change in expression level).

The small sample size may influence the power of the statistical test used and cause type II errors [37]. In other words, the research failed to identify a significant difference and accepted the null hypothesis incorrectly. Furthermore, as mentioned in the MIQE guideline [38], using more than one reference gene for normalization is preferable, which is one of the other possible limitations of this study.

## Conclusion

In conclusion, this study aimed to investigate DECs associated with gastric adenocarcinoma that could be used as future biomarkers and therapeutic targets. Using bioinformatics analysis, we identified 13 up-regulated and 19 down-regulated circRNAs that could be associated with gastric cancer development. Using real-time PCR, we examined variations in the expression of *hsa-circ-0002019* and *hsa-circ-0074736* in plasma samples. These two circRNAs were not found to be differentially expressed in plasma, therefore, they are unlikely to be beneficial as diagnostic biomarkers. However, since they can regulate genes highly associated with patients' overall survival, they may be useful as prognostic biomarkers following further investigation. In addition, we identified important genes associated with GAC using microarray meta-analysis and circRNA-miRNA-mRNA regulatory networks. The potential gene ontologies and pathways associated with DECs were also identified, contributing to a better understanding of the biological processes and possible underlying mechanisms of action of the proposed dysregulated circRNAs in GAC.

## Ethics approval and consent to participate

The study was conducted under the Declaration of Helsinki. All participants signed the informed consent form before donating blood samples. The informed consent and the experiment process were approved by the Research Ethics Committee of the Iran University of Medical Sciences (IR.IUMS.FMD.REC.1399.561).

## Consent for publication

Not applicable.

## Availability of data and materials

The microarray datasets that were analyzed during the current study are available in GEO repository (www.ncbi.nlm.nih.gov/geo). Accession numbers are listed in Table 2.

## Funding

This work was granted by Iran University of Medical Sciences #984416081

## CRediT authorship contribution statement

**Liora Yesharim:** Conceptualization, Writing – original draft. **Saeed Talebi:** Formal analysis, Writing – review & editing. **Marzieh Mojbafan:** Writing – review & editing. **Mahdi Alemrajabi:** Project administration. **Shahram Teimourian:** Supervision, Writing – review & editing.

## Declaration of Competing Interest

The authors declare that they have no known competing financial interests or personal relationships that could have appeared to influence the work reported in this paper.

## Appendix Supplementary materials

Image, application 1
