# Supplementary material for: An evaluation of gastric adenocarcinoma-associated CircRNAs based on microarray meta-analysis and ceRNA networks
Source: Transl Oncol. 2022 Dec 29;28:101611. doi: 10.1016/j.tranon.2022.101611 (PMC9830311; doi:10.1016/j.tranon.2022.101611)
Supplement: Supplementary file 1 [file mmc1.docx]

| Case/ gastric cancer | | |  | Mean Ct value | | |
| --- | --- | --- | --- | --- | --- | --- |
| **Age** | **Gender** | **Pathological Diagnosis** | **reason Sample removed from further analysis** | ***POLR2A (RPII)*** | ***hsa_circ_0002019*** | ***hsa_circ_0074736*** |
| 67 | M | Gastric Adenocarcinoma | Smoker-Turbid plasma-poor RNA quality |  |  |  |
| 57 | M | Gastric Adenocarcinoma |  | 32.45 | 38.65 | 38.95 |
| 45 | M | Gastric Adenocarcinoma |  | 35.42 | 36.00 | 50.00 |
| 71 | F | Gastric Adenocarcinoma |  | 35.19 | 38.52 | 50.00 |
| 75 | M | Gastric Adenocarcinoma |  | 30.21 | 33.56 | 40.00 |
| 64 | M | Gastric Adenocarcinoma |  | 31.53 | 34.24 | 50.00 |
| 57 | M | Gastric Adenocarcinoma |  | 34.85 | 34.66 | 39.90 |
| 67 | M | Gastric Adenocarcinoma | Poor RNA quality |  |  |  |
| 87 | M | Gastric Adenocarcinoma |  | 32.45 | 34.29 | 38.27 |
| 74 | M | Gastric Adenocarcinoma | Poor RNA quality |  |  |  |
| 70 | M | Gastric Adenocarcinoma | Metastasis to lung |  |  |  |
| 70 | F | Gastric Adenocarcinoma | Metastasis to Esophagus |  |  |  |
| 68 | F | Gastric Adenocarcinoma |  | 34.31 | 34.39 | 50.00 |
| 56 | M | Gastric Adenocarcinoma | Smoker-Poor RNA quality |  |  |  |
| 70 | M | Gastric Adenocarcinoma |  | 33.67 | 34.50 | 38.27 |
| 60 | M | Gastric Adenocarcinoma |  | 28.94 | 30.99 | 36.60 |
| 55 | M | Gastric Adenocarcinoma |  | 31.31 | 35.00 | 50.00 |
| 71 | F | Gastric Adenocarcinoma |  | 31.55 | 31.73 | 50.00 |

| **Control/Normal** | |  | **Mean Ct value** | | |
| --- | --- | --- | --- | --- | --- |
| **Age** | **Gender** | **reason Sample removed from further analysis** | ***POL2A (RPII)*** | ***hsa_circ_0002019*** | ***hsa_circ_0074736*** |
| 65 | M | Poor RNA quality |  |  |  |
| 61 | M |  | 33.23 | 34.52 | 50.00 |
| 52 | F |  | 34.62 | 34.04 | 50.00 |
| 62 | M |  | 33.57 | 34.67 | 37.18 |
| 58 | M | Poor RNA quality |  |  |  |
| 73 | F |  | 32.04 | 35.07 | 46.18 |
| 56 | M |  | 32.82 | 33.45 | 37.08 |
| 53 | F |  | 30.59 | 34.22 | 50.00 |
| 74 | M |  | 29.97 | 32.75 | 31.81 |
| 54 | M |  | 31.90 | 33.65 | 33.57 |
| 66 | M |  | 29.10 | 31.98 | 50.00 |
| 53 | F |  | 31.27 | 33.15 | 50.00 |
| 54 | F |  | 30.83 | 32.31 | 36.78 |
| 58 | F |  | 31.79 | 32.74 | 50.00 |
| 69 | M |  | 31.23 | 35.59 | 39.88 |
